# A Rare Case of Intracardiac Extension of Hepatocellular Carcinoma in a Child

**DOI:** 10.1155/2019/4783595

**Published:** 2019-12-05

**Authors:** Abdulrahman Al Jassmi, Hani Humad, Said Abou Eida

**Affiliations:** ^1^Department of Pediatric Hematology/Oncology, Dubai Hospital, PO Box 92227 Dubai, UAE; ^2^Department of Pediatrics, Welcare Hospital, Dubai, UAE

## Abstract

Hepatocellular carcinoma (HCC) is the fifth most common malignancy found in men and ninth most common in women, out of which 72.5% reported cases are from Asia. In children, it accounts for <2% cases worldwide with even rarer incidence of 1.2% involving intracardial extension. However, it presents with a high mortality rate with mean survival ranging from 1 to 4 months. The present case is an extremely rare case of intracardiac extension of HCC in a 3.5-year-old Asian girl with no history of hepatitis B infection presented at an advanced stage of HCC who succumbed within one month of presentation to the hospital.

## 1. Introduction

Historically, first being described in the eighteenth century by Gaspard-Laurent Bayle, hepatocellular carcinoma (HCC) is now the most common malignant liver tumor following hepatoblastoma [1, 2]. Worldwide, it is the third leading cause of cancer-related mortality accounting for 782,000 (95%) deaths out of 782,000 people in 2012 and 781,631 (93%) out of 841,080 people in 2018. It is the fifth most common malignancy found in men and ninth most common in women out of which 72.5% cases are reported from Asia [2, 3]. Although it is quite rare in children, accounting for less than 2% cases worldwide, it presents with high mortality with nearly 80% deaths within the first year of diagnosis [1, 4–7]. In most cases, the diagnosis of HCC is made at an advanced stage with the usual sites of metastases being the lungs, lymph nodes, and bones [8].

Generally, the incidence of cardiac metastasis ranges from 2.3 to 18.3% with a greater propensity of metastases after the fifth decade of life; the most common secondary cardiac tumors are melanoma, mediastinal primary tumors, mesotheliomas, lung carcinomas, breast carcinoma, and renal carcinoma. Albeit found in close proximity to the heart, the intracardiac extension of HCC is extremely rare with an incidence rate of 1.2% [9]. In cardiac metastasis, HCC leads to intracavitary involvement of the right atrium through direct tumor invasion of the inferior vena cava (IVC) causing cardiac symptoms [9, 10].

However, this report is an extremely rare case of intracardiac extension of HCC in a girl with 80% involvement of the right atrium and extension into the right ventricle too, without any cardiac symptoms at the time of presentation.

## 2. Case Report

At the end of November 2012, a 3.5-year-old child presented to the pediatric department with a progressive abdominal swelling for the past two and a half months. Earlier, she was seen in Bangladesh, where antibiotics were prescribed yet she did not take them. Initially, she had an erythematous rash that subsided but had no other symptoms of vomiting, diarrhea, constipation, or difficulty in breathing. On physical examination, the girl has a thin build with an abdominal mass extending from the right hypochondrium downward (8-10 cm) crossing the midline, which had a lobular irregular surface; the upper border of the mass was nonpalpable, and no bruits were present. Chest, cardiovascular, and central nervous system examinations were normal along with the absence of pallor, jaundice, and lymphadenopathy. No signs of edema were noted.

The child was admitted a couple of times for the evaluation of the mass, performing investigations and reaching a final diagnosis. Meanwhile, she was treated symptomatically in the ICU with intravenous hydration and maintaining constant oxygenation. She was followed up conservatively because of the end-staged metastatic HCC and the increased risks of heart surgery-attributable mortality. During this period, she was referred to other centers for investigations, including chest X-ray and angiography and transesophageal echocardiography (TEE) to get insights into not only the location of the mass in relation to the atrial wall or the tricuspid valve but also its position with respect to the superior and inferior vena cava. However, TEE was not performed due to financial constraints. The patient's conditions worsened and she developed fever, periorbital edema, sacral edema, and pedal edema. Her edema worsened, and later she developed difficulty in breathing and had to be put on artificial oxygen to maintain saturation.

**Figure 1:**
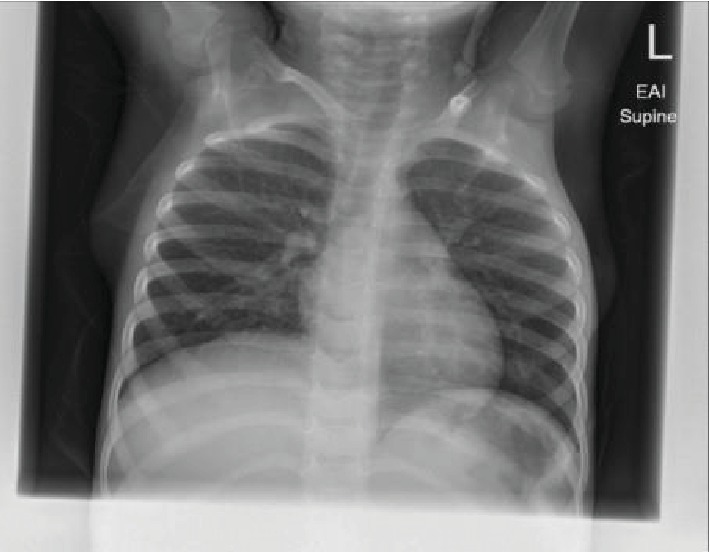
Chest X-ray showing an abdominal mass.

Three months later, cardiac auscultation revealed a sinus heart rhythm and 2/6 (quiet) systolic murmur. Additionally, a bilateral pitting edema was noticed in the lower extremities. Laboratory investigations showed abnormally elevated hemoglobin, platelets, albumin, and hepatic enzymes (Table 1). Additionally, chest X-ray showed an ill-defined infiltrate in the right lower zone with bilateral bronchial wall thickening (Figure 1). Notably, there were no lung metastases. On abdominal ultrasonography, the liver was enlarged with nodular heterogeneous echotexture and multiple echogenic patches without biliary dilatation.

A 2D transthoracic echocardiogram demonstrated a large mass in the right atrium extending to the tricuspid valve without significant obstruction. Computed tomography (CT) of the abdomen showed a large 9.5 × 11.5 × 13.5 cm lobulated, mixed complex solid-cystic mass lesion, which was a predominantly hypodense solid lesion with heterogenous cystic components and intervening thick septa occupying the left lobe of the liver and a large part of the right lobe (segments 5 and 6) and caudate lobe. It extended superiorly above the diaphragm as a well-demarcated 2.5 × 3 cm mass at the cardiophrenic angle dipping into the right atrium of the heart in close contact with IVC. Vessels were stretched rather than compromised or invaded, and portal and hepatic veins were patent. Chest CT showed slight right-sided pleural effusion filling the lateral and posterior costophrenic sulci along with mild pleural effusion on the left side too. A large exophytic mass was noted projecting, as an extension from the extensive liver lesion, into the right atrium. Bone scintigraphy was unremarkable with no evidence of a metastatic lesion.

**Figure 2:**
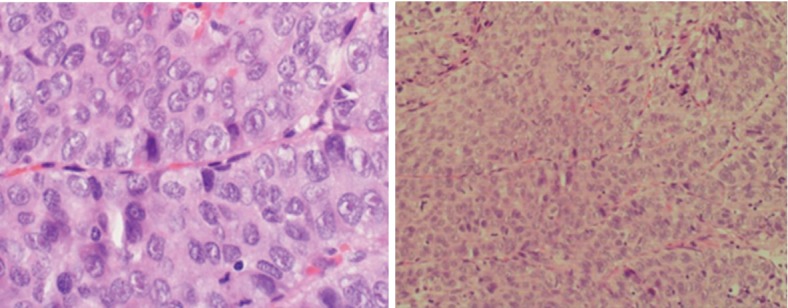
A biopsy showing frequent mitotic figures, pleomorphism, and vesicular nuclei with ill-defined infiltrate in the right lower zone.

Liver biopsy was performed at our institution, and the histopathological report was consistent with a malignant tumor (Figure 2). Immunohistochemistry findings favored the diagnosis of a moderately differentiated HCC. In line with the findings of the physical examination, imaging studies, and histopathological analysis, the patient was finally diagnosed with HCC, which had extended through the inferior vena cava to the right atrium, occupying 80% of the right atrium with an extension to the right ventricle. The Pretreatment Extension of Disease (PRETEXT) system was used for staging, and the tumor was staged as PRETEXT IV, V3.

The patient was readmitted for further management after reaching the final diagnosis of HCC. Her condition was reviewed by a pediatric oncology team and cardiothoracic surgeons, and she was started on chemotherapy with a plan to resect the mass extending to the right atrium. Nevertheless, the patient could not sustain chemotherapy owing to her comorbidities. Unfortunately, the girl died of cardiorespiratory arrest at the operation table during the induction of anesthesia at the end of December 2012. CPR measures were undertaken to resuscitate the child, but the child succumbed to inflow and outflow blockage of the heart.

## 3. Discussion

HCC stages as an abdominal swelling along with discomfort owing to its large size, which is comparable to this case [4]. It is responsible for 23% of all primary pediatric hepatic malignancies with an annual incidence of 0.15 per 100,000 (1975-1995). Overwhelmingly, 75% of children over the age of 10 years are affected; however, it is quite rare in children < 5 years of age, accounting for <10% cases [4, 7]. In contrast to adults, where HCC occurs predominantly on the background of cirrhotic liver, HCC presents in normal livers in the majority (70%) of the pediatric population [11]. Generally, these children are positive for hepatitis B serum antigen (HBsAg), which is in contrast to this case [6]. Other etiological factors related to HCC in children constitute congenital and metabolic conditions including biliary atresia, hereditary tyrosinemia type 1, congenital hepatic fibrosis, and progressive familial intrahepatic cholestasis [7].

In the orient and sub-Saharan African regions, about 75-92% reported cases are positive for serum alpha-fetoprotein level (sAFP). AFP levels are of prognostic value as elevated AFP titers are associated with 1.8 times higher mortality [6]. HCC also carries poor prognosis owing to its chemoresistant nature with an estimated cure rate of only 25-30% [4, 5]. Advanced stages of HCC have a median survival rate of 4-7 months [12]. Tumor resection is the sole curative treatment, and it can be performed either via partial liver resection or hepatic transplantation. In the recent studies, the overall five-year survival rate ranges between 50% and 60% following surgical resection, yet it reaches only 20-30% in pediatric patients with advanced disease at diagnosis who received a combination of surgical resection and chemotherapy [13–15].

Unfortunately, a very large number of pediatric patients have advanced or metastatic HCC at the time of diagnosis, with the lung being the most common site for extrahepatic metastasis [7, 8]. However, an extension of HCC into the right atrium is possible, albeit it is extremely rare with studies indicating 1.2-4.1% of extrahepatic cardiac metastasis [16–19]. An autopsy case study of 439 HCC cases showed 18 cases of right atrium extension of tumor and 5 cases of extension into the right ventricle [20]. HCC has a greater propensity for vascular invasion with portal vein thrombosis being the most common accounting for 20-65% cases, followed by systemic venous thromboembolism at 6% and hepatic vein thrombosis at 1.4-4.9%. However, the tumor thrombus invasion of the inferior vena cava is quite rare accounting for 0.67-3% of all cases [21].

Cardiac metastases occur either via direct extension of a tumor thrombus through IVC invasion or through the bloodstream or the lymphatic system; the most common pathway for HCC is IVC [9, 16]. Isolated cardiac metastasis of HCC is extremely rare, and a literature search shows 17 such cases, none of which were pediatric patients [16, 22].

The frequent location of an intracardiac primary or metastatic tumor is usually the left ventricle and left atrium, with the right ventricle and right atrium being less common. Right atrium tumors may obstruct the orifice or damage the tricuspid valve leading to tricuspid stenosis or regurgitation causing right heart failure with resulting signs and symptoms of dyspnea and lower extremity edema [23]. Great heterogeneity is seen in patients with intracardiac extension of HCC, from being asymptomatic to heart failure, sudden cardiac arrest, Budd-Chiari syndrome, and pulmonary embolism [24]. The reported patient had no cardiac symptoms at the time of presentation, until at the later stage she developed fever and periorbital, sacral, and pedal edema along with difficulty in breathing. The prognosis for intracardiac metastasis of HCC is dismal with mean survival ranging from 1 to 4 months, irrespective of treatment [12, 25].

Cardiac metastasis is best appreciated in transesophageal echocardiography (TEE), transthoracic echocardiography (TTE) along with CT scan, and MRI, which form the mainstay for diagnosing HCC metastasis to the heart. These help in identifying the extension of the tumor to the heart, right ventricular outflow obstruction, pulmonary thromboembolism, and pericardial effusion [18]. The only effective treatment option includes surgical extraction of the thrombus along with tumor resection, although patient management with advanced HCC with intracardiac extension is difficult and extremely risky. Other management options include transarterial chemoembolization (TACE), ablation, radiation, and chemotherapy [19, 24, 26]. Although the patient in the present report was opted for surgical excision of the tumor, the patient succumbed to inflow and outflow blockage of the heart during anesthesia initiation at the operation table. She died in <1 month after being presented to the hospital.

HCC with cardiac metastasis has very poor prognosis, more so, in pediatric patients. The present case is an extremely rare case of hepatocellular carcinoma extension to the right atrium along with extension to the right ventricle in a 3.5-year-old girl. This is the first literature review involving a child with intracardiac extension of HCC. Awareness in clinicians about such rare cases will affect the overall diagnosis and management of patients.

## Figures and Tables

**Table 1:** The results of the laboratory investigations of the patient at different intervals.

Tests	Normal range	Result 1	Results 2	Results 3
Hemoglobin (g/dL)	11-13	15.7^∗^	16.4^∗^	14^∗^
White blood cells (×10^3^/*μ*L)	5.0-15.0	14.2	11	14.9
Platelets (×10^3^/*μ*L)	150-450	523^∗^	489^∗^	290
Albumin (g/dL)	3.8-5.4	3.9	3.6^∗^	2.5^∗^
Alanine aminotransferase (U/L)	0-33	29	48^∗^	1573^∗^
Aspartate aminotransferase (U/L)	<281	242	310^∗^	199
HIV AG/AB	Negative	Negative	—	—
Alpha-fetoprotein (ng/mL)	0.5-5.5	—	—	>30,000^∗^
Urinalysis		Calcium oxalate crystals: 3+ White blood cells: 0-5		
